# Prenatal Lead Exposure in Mice: Age-Related, Sex-Specific Effects Observed

**DOI:** 10.1289/ehp.116-a129b

**Published:** 2008-03

**Authors:** Julia R. Barrett

Children with low-level prenatal lead exposure may suffer reduced cognitive function, impaired motor ability, and visual and auditory processing problems. Other effects, such as accelerated age-related functional decline or delayed neurotoxicity, may become apparent in adulthood, though few studies have examined the long-term consequences of exposure. A novel animal model now reveals age-related, male-specific, and nonmonotonic dose–response effects associated with low-level prenatal lead exposure **[*EHP* 116:355–361; Leasure et al.]**.

To model gestational lead exposure (GLE), one group of dams received tap water or drinking solutions containing low (27 ppm), moderate (55 ppm), or high (109 ppm) concentrations of lead beginning 2 weeks before mating and continuing until postnatal day 10. To measure postnatal lead exposure (PLE), another group of dams received tap water or water that contained low or moderate levels of lead from birth to weaning. The offspring of both groups were measured at birth and several times throughout the following year for weight and blood lead concentrations.

Blood lead levels ranged from 10 μg/dL or less in low-exposure GLE offspring to 42 μg/dL in the high-exposure GLE group at post-natal day 10, and from 10 μg/dL in low-exposure PLE offspring to 27 μg/dL in the high-exposure PLE group at postnatal day 21. By postnatal day 30 for GLE offspring and postnatal day 60 for PLE offspring, blood lead levels were no different than in controls.

At 1 year of age, male and female offspring were assessed for exploratory activity and interlimb balance and coordination. Because human developmental studies have demonstrated that males are at higher risk of deficits related to early lead exposure, additional testing of male offspring included measuring fore-brain and striatal levels of dopamine and its major metabolite, 3,4-dihydroxyphenylacetic acid.

Lead exposure did not affect body weight of any PLE mice or of female GLE mice, but there was a significant inverse relationship between lead exposure and body weight for male low-exposure and high-exposure GLE mice (26% and 13% heavier than controls, respectively). Male GLE mice also exhibited significantly less exploratory activity, with a greater effect again seen in the low-exposure group. This group also had significantly poorer balance and coordination, lower forebrain levels of dopamine, and higher forebrain and striatal levels of 3,4-dihydroxyphenylacetic acid.

Multiple endocrine or metabolic mechanisms that occurred during gestational development could explain the late-onset obesity observed at 1 year of age. The diminished exploratory activity may reflect an altered stress response arising from lead-related changes in the hypothalamic–pituitary–adrenal axis and dopaminergic systems. Changes in the forebrain may also have implications for attention deficit/hyperactivity disorder, as abnormalities in the prefrontal cortex appear to underlie the disorder. Multidisciplinary behavioral, biochemical, and molecular studies are needed to test and explain the reported novel findings, which highlight the need for realistic lifetime dose–response assessment of toxicants.

